# Factors Associated With Emergency Department Use by Patients With and Without Mental Health Diagnoses

**DOI:** 10.1001/jamanetworkopen.2018.3528

**Published:** 2018-10-19

**Authors:** Matthew J. Niedzwiecki, Pranav J. Sharma, Hemal K. Kanzaria, Shannon McConville, Renee Y. Hsia

**Affiliations:** 1 Mathematica Policy Research, Oakland, California; 2 Philip R. Lee Institute for Health Policy Studies, University of California, San Francisco; 3 Department of Emergency Medicine, University of California, San Francisco; 4 Alpert Medical School, Brown University, Providence, Rhode Island; 5 Public Policy Institute of California, San Francisco

## Abstract

**Importance:**

An association between frequent use of the emergency department (ED) and mental health diagnoses is frequently documented in the literature, but little has been done to more thoroughly understand why mental illness is associated with increased ED use.

**Objective:**

To determine which factors were associated with higher ED use in the near future among patients with and without mental health diagnoses.

**Design, Setting, and Participants:**

A retrospective case-control study of all patients presenting to the ED in California in 2013 using past ED data to predict future ED use. Data from January 1, 2012, through December 31, 2014, from California’s Office of Statewide Health Planning and Development were analyzed.

**Main Outcomes and Measures:**

Factors associated with higher ED use in the year following an index visit for patients with vs without a mental health diagnosis.

**Results:**

Among the 3 446 338 individuals in the study (accounting for 7 678 706 ED visits), 44.6% (1 537 067) were male; 31.6% (1 089 043) were between the ages of 18 and 30 years, 40.3% (1 338 874) were between the ages of 31 and 50 years, and 28.1% (968 421) were between the ages of 51 and 64 years. The mean (SD) number of ED visits per patient per year was 1.69 (2.56), and 29.1% of patients (1 002 884) had at least 1 mental health diagnosis. Previous hospitalization and high rates of lagged ED visits were associated with higher future ED use. The severity of the mental health diagnosis (mild, moderate, or severe) was associated with increased ED visits (incidence rate ratio [IRR], 1.029; 95% CI, 1.02-1.04 for mild; IRR, 1.121; 95% CI, 1.11-1.13 for moderate; and IRR, 1.226; 95% CI, 1.22-1.24 for severe). Little evidence was found for interaction effects between mental health diagnoses and other diagnoses in predicting increased future ED use.

**Conclusions and Relevance:**

Certain classes of mental health diagnoses were associated with higher ED use. The presence of a mental illness diagnosis did not appear to interact with other patient-level factors in a way that meaningfully altered associations with future ED use.

## Key Points

**Question:**

What factors are associated with higher emergency department (ED) use among patients with and without mental health diagnoses?

**Findings:**

This case-control study found that previous hospitalization and high rates of lagged ED visits were associated with higher future ED use. Mild, moderate, and severe mental health diagnoses were associated with increases of 2.9%, 12.1%, and 22.6%, respectively, in ED use.

**Meaning:**

Prior patient visit patterns and patient illness severity associated with mental health diagnoses could be important contributors to increased ED use.

## Teaser

This case-control study examines factors associated with higher emergency department (ED) use among US patients with vs without mental health diagnoses.

## Introduction

The rate of visits to US emergency departments (EDs) continues to rise, and EDs increasingly treat medically underserved patients.^1^ Because EDs are a high-cost care setting, policymakers have concentrated on ways to reduce potentially avoidable utilization.^2,3^ One area of focus has been on better understanding the profile of frequent ED users, as they account for a disproportionate percentage of ED visits and expenditures. In fact, 4% to 8% (15.7 million) of frequent ED users account for 18% to 30% of total ED visits.^4,5,6^

Approximately 50% of frequent ED users have a mental health diagnosis,^4^ and this group has higher rates of morbidity and mortality and incurs higher medical costs over time.^7,8,9^ Additionally, frequent ED users often have many issues that historically have been considered nonmedical, including homelessness, food insecurity, and addiction.^7^ Nevertheless, up to 80% of patients with mental illness seek care in medical—instead of behavioral care—settings, where they often leave without treatment for mental illness.^10^ Given the potentially low efficacy of ED-based treatment for mental illness, identifying characteristics related to increased ED use among patients with mental illness is an important step toward improving their health care access, quality, and costs.

Recent health policy innovations have focused on patients with mental health conditions given their complex needs and high cost burden.^11,12^ At the federal level, the Center for Medicare & Medicaid Services has several initiatives currently supporting states to provide more coordinated care and improved case management for high-utilizing patients with public insurance. With California’s expansion of its Medicaid program under the Affordable Care Act in 2014, a new set of mental health benefits for Medicaid members with mild-to-moderate mental health needs were added and delegated to Medicaid managed care plans.^13^ County mental health plans, however, remain responsible for providing care to Medicaid beneficiaries with severe mental health needs.

To help inform such efforts, it is important to understand factors associated with increased ED use among patients with mental health conditions and also to recognize how these factors differ in patients without mental illness, if at all. To our knowledge, no prior studies have examined how factors associated with increased future ED utilization differ between patients with and without mental health diagnoses and to what extent the severity of mental health needs is associated with future ED usage. In our study, we used regression analysis to estimate which medical and social factors are associated with future ED utilization and how they differ between those with and without mental health diagnoses.

## Methods

We performed a retrospective analysis to predict ED use in the year following an index visit by using observable patient characteristics available in the previous year’s ED records for patients who presented to a California acute care hospital in 2013. We adhered to the Strengthening the Reporting of Observational Studies in Epidemiology (STROBE) reporting guideline in the reporting of our results and discussion.^14^ This study used deidentified, precollected data, and was therefore deemed exempt from review by the University of California, San Francisco, institutional review board.

### Setting and Study Population

We used nonpublic data from January 1, 2012, through December 31, 2014, from California’s Office of Statewide Health Planning and Development (OSHPD), which provides detailed information on all ED visits at licensed hospitals in the state. Importantly, the data set is a census of all nonfederal hospitals in California and all payers, not just a subset of hospitals or payers; such an approach is critical to accurately and comprehensively capture ED use among patients who may visit multiple EDs and change insurance status frequently.^15^ Hospitals report data to OSHPD and perform routine accuracy checks using OSHPD’s Medical Information Reporting for California online system to reduce the likelihood of potential reporting errors and missing data.^16^ For each patient, the first ED visit in 2013 was considered his or her index visit. Using data from all ED visits in the 365 days prior to the index visit, we built a regression model to predict ED use, including inpatient hospitalizations originating from the ED, at any California hospital over the following year. For example, for a patient whose first visit was on January 1, 2013, we included data on that patient from January 1, 2012, through December 31, 2012, along with information from the index visit, as the look-back period, and we included data going forward through January 1, 2014, as the follow-up period. We used a unique record linkage number (RLN) to track each patient’s utilization over time. We defined and classified mental health conditions using the primary category of “mental health” by the Healthcare Cost and Utilization Project’s (HCUP) Clinical Classifications Software (CCS).^17^

**Figure 1.**
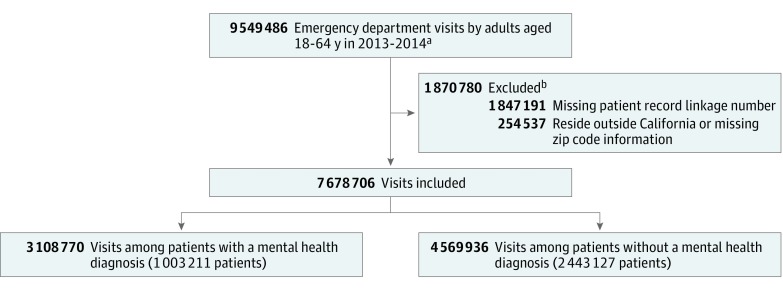
Flow Diagram of Emergency Department Visits From Look-Back Period ^a^Visits within the 365-day look-back period by patients aged 18 to 64 years with at least 1 mental health diagnosis and at least 1 emergency department visit in 2013. Index visit included in count. ^b^Visits may have met more than 1 exclusion criterion.

We focused on nonelderly adults, excluding individuals aged younger than 18 years or older than 64 years. We excluded patients who did not have a valid RLN, did not have a valid zip code, or did not live in California (eTable 1 in the Supplement includes a comparison of visits with and without a valid RLN). The final sample included 7 678 706 ED visits by 3 446 338 patients during the 365-day look-back period, including the index visit (Figure 1).

### Primary Outcome

We examined the association between observable patient characteristics based on ED visits from the 365 days prior to the index visit and the number of ED visits in the year following the index visit.

### Covariates

The key covariates of interest were related to a patient’s mental health diagnoses in the year prior to the index ED visit. We included an indicator for whether the patient ever had a primary mental health diagnosis as well as indicators for mental health illness severity based on the HCUP-derived *Severity-of-Illness Classification for Mental and Substance-Use Disorders.*^18^ The algorithm grouped *International Classification of Diseases, Ninth Revision, Clinical Modification* (*ICD-9-CM*) diagnosis codes into 3 ordinal categories of severity: mild (eg, social phobia and alcohol abuse), moderate (eg, panic disorder and drug abuse), and severe (eg, psychoses and drug dependency). These factors are associated with more frequent hospitalization (higher severity is associated with a higher risk of future hospitalization) and can be used with OSHPD discharge data (eTable 2 in the Supplement).

We also included an indicator for each of the non–mental health–related CCS multilevel diagnosis groups.^17^ We controlled for patient age, sex, race, and insurance status; whether the patient was ever admitted to the hospital during the 365 look-back period; the poverty rate in the patient’s zip code; and whether the patient lived in an urban or rural county. Patient zip code of residence, which determined poverty and urban designation, was based on the modal zip code of all patient visits in the look-back period. We also controlled for the number of ED visits in the prior 365 days. Other studies have found these covariates to be associated with frequent ED use.^4,19,20,21^ The insurance status variables controlled for whether the patient was ever uninsured or covered by public insurance with the reference group being patients who remained privately insured throughout the study period.

In addition, we estimated a model in which we interacted a mental health diagnosis indicator variable with all other covariates to determine whether associations differ between certain factors and future ED use depending on whether patients have mental health diagnoses. While it has been shown that mental health diagnoses are associated with substantially larger costs,^22^ whether mental illness is an independent cost or whether mental health diagnoses interact with other diseases in a way that amplifies a patient’s need for treatment remains unknown. The interaction terms in our model allow us to examine whether the presence of mental health diagnosis amplifies (positive interaction coefficients) or dampens (negative interaction coefficients) other factors in the model.

By including the prior year’s utilization in our predictive model, the other covariates are interpreted as predictors of future use conditional on prior utilization levels. In this context, variables such as diagnoses, insurance status, or poverty status can be interpreted as factors contributing to the persistence or decline of ED use relative to the baseline level over time.

In a sensitivity analysis, we excluded patients with the top 0.001% and 0.01% of total visits (greater than 191 and 87 visits, respectively) in the year following the index visit to remove the effect of extreme outliers.

### Statistical Analysis

We used a negative binomial regression model with a log-link function to study the association between the number of ED visits in a 1-year period and the patient’s observable characteristics from the prior year. The negative binomial regression model was used because the dependent variable in our regression was count of ED visits. We reported incidence rate ratios (IRRs), which indicate the association between an independent variable and the relative frequency of the outcome (ED visits) in the 1-year follow-up period. Two-sided significance tests were used with a significance level of .05. Standard errors are robust to heteroskedasticity. There was no need for clustering the standard errors because each observation represents a single patient and not a visit. Stata statistical software version 13 (StataCorp) was used for all statistical analysis.^23^

## Results

**Figure 2.**
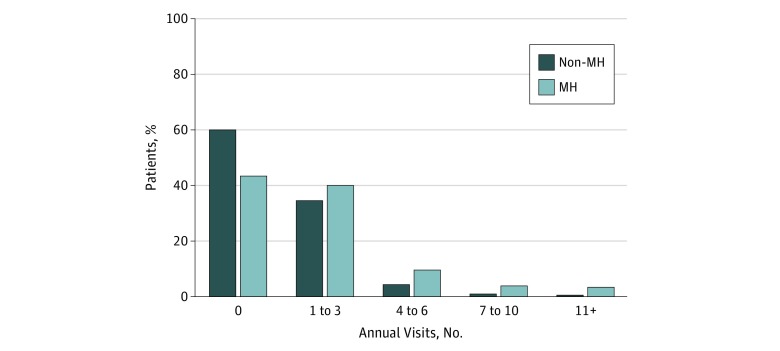
Distribution of Patient Visits by Presence of Any Mental Illness MH indicates mental health.

There were 3 446 338 individuals who accounted for 7 678 706 ED visits in the study. Among study participants, 44.6% (1 537 067) were male; 31.6% (1 089 043) were aged 18 to 30 years, 40.3% (1 338 874) were between the ages of 31 and 50 years, and 28.1% (968 421) were aged 51 to 64 years (Table 1). Table 1 shows additional descriptive characteristics of our sample. The mean (SD) number of ED visits per patient per year was 1.69 (2.56), and 29.1% of patients (1 002 884) had at least 1 mental health diagnosis. Figure 2 shows the distribution of visit counts over a 365-day period for patients with and without mental health diagnoses. Compared with patients without a mental health diagnosis, those with a mental health diagnosis generally had higher visit volumes. Among those with a mental health diagnosis, those with 4 or more ED visits, or 16.8% of patients, made up 39.6% of the total visits.

**Table 1.** Descriptive Characteristics of Study Sample

Characteristics	% (SE)^a^
Age, y	
18-30	31.6 (0.14)
31-40	20.0 (0.02)
41-50	20.3 (0.02)
51-64	28.1 (0.02)
Male	44.6 (0.03)
Race/ethnicity	
White	45.3 (0.03)
Black	12.1 (0.02)
Hispanic	33.1 (0.03)
Other	6.2 (0.01)
Unknown	7.2 (0.01)
Insurance status	
Privately, always	46.1 (0.03)
Medicare, ever	7.2 (0.01)
Medicaid, ever	21.9 (0.02)
Uninsured, ever	28.3 (0.02)
Urban county of patient residence	97.1 (0.01)
Visit characteristics	
Ever admitted inpatient	4.7 (0.01)
Any primary mental health diagnosis	29.1 (0.02)
Non–mental health Clinical Classifications Software codes^b^	
Infectious and parasitic diseases	11.1 (0.02)
Neoplasms	4.0 (0.01)
Endocrine, nutritional, and metabolic diseases and immunity disorders	25.2 (0.02)
Diseases of the blood and blood-forming organs	6.9 (0.01)
Diseases of the nervous system and sense organs	24.2 (0.02)
Diseases of the circulatory system	29.1 (0.02)
Diseases of the respiratory system	22.5 (0.02)
Diseases of the digestive system	21.8 (0.02)
Diseases of the genitourinary system	18.9 (0.02)
Complications of pregnancy, childbirth, and the puerperium	5.6 (0.01)
Diseases of the skin and subcutaneous tissue	8.2 (0.01)
Diseases of the musculoskeletal system and connective tissue	20.1 (0.02)
Congenital anomalies	0.7 (0.00)
Certain conditions originating in the perinatal period	0.0 (0.00)
Injuries and poisoning	32.1 (0.03)
Symptoms, signs, and ill-defined conditions and factors influencing health status	32.1 (0.03)
Residual codes, unclassified, all E codes and V codes	31.6 (0.03)

^a^ Standard error of the mean is expressed in units of percentage points.

^b^ Diagnoses are grouped according to the Healthcare Cost and Utilization Project’s Clinical Classifications Software and represent any diagnosis recorded in any visit to the emergency department or inpatient hospitalization following an emergency department visit.

Table 2 shows results from negative binomial regression reported as IRRs, where the dependent variable is a count of ED visits over the 1-year period following the index visit. An IRR of 1.20, for example, indicates that a 1-unit change in the independent variable is associated with a 20% increase in ED visits in the year after the index visit. One of the factors most strongly associated with increased ED use in the next year was whether the patient was admitted to any hospital in the previous year (IRR, 4.88; 95% CI, 4.83-4.93), followed by high volume of ED visits in the previous year (IRR, 1.64; 95% CI, 1.62-1.66 for those with 4-10 visits vs IRR, 1.97; 95% CI, 1.86-2.08 for those with 11-17 visits and IRR, 5.91; 95% CI, 5.00-6.98 for those with ≥18 visits in the prior year). Other nonclinical factors, including insurance coverage and neighborhood poverty, also had a significant association with future ED visits. Patients with Medicaid (IRR, 1.939; 95% CI, 1.93-1.95), Medicare (IRR, 1.812; 95% CI, 1.79-1.83), or no insurance (IRR, 1.612; 95% CI, 1.60-1.62) in the previous year, had more visits in the following year compared with those with private insurance. Patients who lived in moderately poor zip codes (middle tercile for median zip code income) were predicted to have 11.1% (95% CI, 11.0%-11.2%) more visits in the following year, and patients who lived in the poorest zip codes (lowest tercile for median zip code income) had 11.7% (95% CI, 11.6%-11.7%) more visits in the following year, both relative to patients who lived in zip codes with the highest income group.

**Table 2.** Regression Analysis of Study Sample^a^

Variables	IRR (95% CI)
Patient characteristics (N = 3 446 338)	
Insured	
Privately, always	1 [Reference]
Medicare, ever	1.81 (1.79-1.83)^b^
Medicaid, ever	1.94 (1.93-1.95)^b^
Uninsured, ever	1.61 (1.60-1.62)^b^
Poverty in patient’s zip code	
Low	1 [Reference]
Medium	1.11 (1.10-1.12)^b^
High	1.17 (1.16-1.17)^b^
Visit characteristics	
Infrequent user (0-3 visits) in prior y	1 [Reference]
Frequent user in prior y	
4-10 visits	1.64 (1.62-1.66)^b^
11-17 visits	1.97 (1.86-2.08)^b^
≥18 visits	5.91 (5.00-6.98)^b^
Primary MH diagnosis	1.31 (1.30-1.32)^b^
Admitted inpatient	4.88 (4.83-4.93)^b^
Diagnoses	
Mental illness	1.26 (1.22-1.29)^b^
MH diagnosis (Healthcare Cost and Utilization Project)	
Mild	1.03 (1.02-1.04)^b^
Moderate	1.12 (1.11-1.13)^b^
Severe	1.23 (1.22-1.24)^b^
Residual codes (omitted)	
Infectious and parasitic diseases	1.00 (1.0-1.01)
Neoplasms	1.04 (1.03-1.05)^b^
Endocrine, nutritional, and metabolic diseases and immunity disorders	0.99 (0.99-1.0)^c^
Diseases of the blood and blood-forming organs	0.83 (0.82-0.84)^b^
Diseases of the nervous system and sense organs	1.22 (1.21-1.22)^b^
Diseases of the circulatory system	1.06 (1.05-1.06)^b^
Diseases of the respiratory system	1.22 (1.21-1.23)^b^
Diseases of the digestive system	1.13 (1.12-1.14)^b^
Diseases of the genitourinary system	1.19 (1.18-1.19)^b^
Complications of pregnancy, childbirth, and the puerperium	1.04 (1.03-1.05)^b^
Diseases of the skin and subcutaneous tissue	1.33 (1.32-1.34)^b^
Diseases of the musculoskeletal system and connective tissue	1.25 (1.24-1.25)^b^
Congenital anomalies	0.98 (0.95-1.00)^d^
Certain conditions originating in the perinatal period	0.98 (0.82-1.18)
Injury and poisoning	0.98 (0.98-0.99)^b^
Symptoms, signs, and ill-defined conditions	1.17 (1.16-1.17)^b^
Interactions	
MH × Medicare insured, ever	0.84 (0.83-0.85)^b^
MH × Medicaid insured, ever	0.87 (0.87-0.88)^b^
MH × uninsured, ever	0.93 (0.92-0.94)^b^
MH × medium poverty in patient's zip code	0.97 (0.96-0.98)^b^
MH × high poverty in patient's zip code	0.96 (0.94-0.97)^b^
MH × frequent user (4-10 visits) in prior y	0.68 (0.67-0.69)^b^
MH × frequent user (11-17 visits) in prior y	0.62 (0.59-0.66)^b^
MH × frequent user (≥18 visits) in prior y	0.54 (0.45-0.64)^b^
MH × admitted inpatient	0.68 (0.67-0.69)^b^
MH × infectious and parasitic diseases	1.01 (1.00-1.03)^c^
MH × neoplasms	0.93 (0.91-0.94)^b^
MH × endocrine, nutritional, and metabolic diseases and immunity disorders	0.93 (0.92-0.94)^b^
MH × diseases of the blood and blood-forming organs	1.00 (0.99-1.02)
MH × diseases of the nervous system and sense organs	1.04 (1.03-1.05)^b^
MH × diseases of the circulatory system	0.99 (0.98-1.00)^b^
MH × diseases of the respiratory system	0.97 (0.96-0.98)^b^
MH × diseases of the digestive system	1.03 (1.02-1.03)^b^
MH × diseases of the genitourinary system	0.94 (0.93-0.95)^b^
MH × complications of pregnancy, childbirth, and the puerperium	1.06 (1.04-1.08)^b^
MH × diseases of the skin and subcutaneous tissue	0.88 (0.87-0.89)^b^
MH × diseases of the musculoskeletal system and connective tissue	1.00 (0.99-1.01)
MH × congenital anomalies	1.03 (0.99-1.07)
MH × certain conditions originating in the perinatal period	0.93 (0.73-1.19)
MH × injury and poisoning	1.15 (1.14-1.16)^b^
MH × symptoms, signs, and ill-defined conditions	1.12 (1.11-1.13)^b^

Abbreviations: IRR, incidence rate ratio; MH, mental health.

^a^ Negative binomial regression analysis (log link function) with an outcome variable indicating the total count of emergency department visits in the 365 days following the index visit. Diagnoses are grouped according to the Healthcare Cost and Utilization Project’s Clinical Classification Software and represent any diagnosis recorded in any visit to the emergency department or inpatient hospitalization following an emergency department visit. The omitted diagnosis group is residual codes, unclassified, and E codes (Clinical Classification Software category 18).

^b^ *P* < .01.

^c^ *P* < .05.

^d^ *P* < .10.

Mental health conditions were associated with the second largest increase in ED use, second only to diseases of the skin and subcutaneous tissue. A mental health diagnosis in the prior year was associated with more ED visits in the following year (IRR, 1.256; 95% CI, 1.22-1.29) compared with patients without a mental health diagnosis. Moreover, an ED visit with a primary discharge diagnosis related to mental health in the previous year was associated with more ED visits in the following year (IRR, 1.309; 95% CI, 1.30-1.32) compared with patients with only a secondary mental health discharge diagnosis. More severe mental health diagnoses were associated with more ED visits in the following year. Diagnoses classified as mild, moderate, and severe was associated with increased ED visits (IRR, 1.029; 95% CI, 1.02-1.04 for mild; IRR, 1.121; 95% CI, 1.11-1.13 for moderate; and IRR, 1.226; 95% CI, 1.22-1.24 for severe). Other conditions associated with large increases or decreases in future visits included diseases of the blood and blood-forming organs (IRR, 0.83; 95% CI, 0.82-0.84), diseases of the nervous system and sense organs (IRR, 1.22; 95% CI, 1.21-1.22), diseases of the respiratory system (IRR, 1.22; 95% CI, 1.21-1.23), and diseases of the skin and subcutaneous tissue (IRR, 1.33; 95% CI, 1.32-1.34).

We also sought to examine how the presence of mental health conditions may or may not interact with other factors. The interaction effect with a mental health diagnosis was relatively small in magnitude and in most cases was negative. A mental health diagnosis was associated with 3.32 times as many ED visits in following year if a patient was hospitalized at some point in the prior year, whereas no mental health diagnosis was associated with 4.88 times as many visits if the patient was hospitalized in the prior year, all other factors held constant. Small negative interaction effects were found for insurance status, poverty measures, and diseases of the skin and subcutaneous tissue as well. One prominent amplification effect was noted for injury and poisoning diagnoses (including both unintentional and intentional self-injury and self-poisoning), which, when combined with a mental health diagnosis, were associated with an additional 1.13 times as many ED visits in the following year in addition to the independent association with a mental health diagnosis. An injury or poisoning diagnosis with no mental health diagnosis was associated with only 0.98 (95% CI, 0.98-0.99) times as many visits in the following year.

Results were not sensitive to censoring patients whose visit counts were large outliers. The regression coefficients were unchanged in sign or significance and only slightly different in magnitude (available on request). We also tested sensitivity to using a logistic regression model in which the outcome was frequent ED use (≥4 visits in the 1-year follow-up) rather than using a continuous count of ED visits (eTable 3 in the Supplement). We found that the same coefficients—high levels of ED use in the prior year, hospitalization, and mental health diagnosis—emerged as significant and strongly associated with the outcome variable. Finally, we tested sensitivity of our results to an alternative categorization based on the HCUP severity index (eTable 4 in the Supplement). In the alternative specification, we required a patient to have at least 2 diagnoses in a given mental health category (mild, moderate, and severe) to be classified in that category. We did this to avoid potential misclassification due to a single diagnosis. Under the alternative specification, we found that the IRRs for each category remained larger than 1 and slightly larger in magnitude, although the monotonic relationship between increased severity and increased ED visits no longer held.

## Discussion

To our knowledge, this is the first study to use the full census of ED visits in a large, diverse state to specifically evaluate what factors related to mental health diagnoses are associated with future ED use. The locus of factors associated with ED use, while not necessarily causal in our analysis, suggest that prior patient visit patterns and patient illness severity could be important contributors to increased ED use. Consistent with previous literature, we found that clinical factors related to illness severity were important factors associated with future ED use, and high utilization in the prior year persisted and predicted higher utilization in the following year.^24^

We also found that nonclinical factors matter; Medicaid coverage and lack of insurance were associated with greater ED use than continuous private insurance.^6,7,21,25^ We also found that Medicare coverage, which in the nonelderly population aged 18 to 64 years is synonymous with being disabled, was associated with more utilization and had a similar association as Medicaid coverage. Living in a high-poverty area was also associated with higher ED use compared with living in low-poverty areas, even after controlling for patient demographics, insurance, and clinical characteristics. For individuals who live in poor neighborhoods and have limited access to outpatient services because they are uninsured or underinsured, the ED remains a safety net for access to health care services.^1,26,27,28^

Specific diagnosis groups, particularly mental health diagnoses and skin disease, were also associated with higher rates of ED use in the future. As other studies have also found, a primary diagnosis of mental health was associated with a higher rate of future use than a secondary diagnosis.^29,30,31,32^ Furthermore, as the severity of the mental health diagnosis increased, there was an increase in the associated number of future ED visits. Combined, a patient with a primary mental health diagnosis and a severe mental health diagnosis like schizophrenia would be predicted to have more than 2 times as many visits compared with a patient without any mental health diagnoses, holding other factors such as number of prior ED visits and insurance constant. We found a consistent increase in the rate of ED visits as mental illness severity increased from mild to moderate to severe and we did so in a forward looking, predictive model that controls for lagged ED utilization and uses count data rather than a retrospective model with binary outcomes.

This is particularly salient in California because the state’s Medicaid program has instituted major changes in how it delivers and pays for mental health services. Our results suggest there is room to improve care coordination, particularly for individuals with severe mental health diagnoses. Although our study focuses on California, this work is also relevant to efforts to integrate physical and mental health nationwide.

Perhaps surprisingly, we found little evidence for positive interaction effects between mental health diagnoses and other illnesses in predicting increased future ED use. In general, the interaction effects between mental health and other diagnoses were negative, suggesting that, at least when it comes to ED visits, mental health diagnoses do not interact other diagnoses to increase utilization. Future work, perhaps using more integrated data sources with information on outpatient utilization and prescription drug use, is warranted and could provide additional insights into the interaction between mental health diagnoses and other diagnoses in predicting health care services use.

As health care systems continue to evolve under the Affordable Care Act and other major policy changes intended to increase access to treatment for patients with behavioral health conditions, it is critical to monitor utilization patterns of individuals with mental health needs to better understand how to provide high-quality care in the most appropriate setting.^33,34^ Given that such a large share of health care costs is paid for by the public sector (with Medicaid being the largest single payer of mental health services in the country) it is important that delivery systems, health plans, and public agencies continue to work toward high-quality, cost-conscious models of care.^35^

Continuity and coordination of care critical for effective mental health care treatment is lacking in public health delivery systems.^36,37^ Case management for frequent ED users can reduce potentially avoidable ED use in a cost-effective manner.^8,38^ Expanding access that is consistent, high quality, and coordinated across medical health, mental health, and social services is needed to improve care for such patients.^39,40,41^

### Limitations

First, because we are using discharge diagnoses, we only observe patients who visit the ED at least once and cannot observe those who have never visited the hospital. The comparison group therefore does not include patients who do not visit the ED. Second, while the nonpublic OSHPD data are a comprehensive data set of all nonfederal acute care hospitals in California, we do not have information about visits to hospitals located out of state. The database, which includes the entire population for a large, diverse state, remains a strength of the analysis. Third, we base our diagnosis information on ED encounters only and do not have access to diagnoses made by other physicians in primary care or outpatient mental health settings. As with all administrative data, there are limitations to OSHPD. Administrative data may not capture disease severity, and while several diagnoses may be linked to a visit, it may be unclear which one is truly driving utilization. While our data contain up to 24 secondary *ICD-9* codes per visit, it is possible that certain diagnoses listed are not relevant to a given visit. For example, if 1 visit lists 5 diagnoses, only 1 may be the current reason for the patient’s ED visit, while the others may be background comorbidities not relevant to the current visit. If all historical diagnoses are included in the coding of the current visit (which may increasingly happen given autopopulation from electronic health records), it then becomes unclear from the administrative data which diagnoses are relevant for this particular visit. Alternatively, administrative data may also have missing diagnoses (eg, a frequent ED user who presents often with the diagnosis of chronic foot pain that may have an underlying undiagnosed mental illness driving the utilization). If they do not seek outpatient care, then it will be much less likely that a mental health condition will be linked with the visit (and, therefore, the patient). It is possible that mental health diagnoses may be undercaptured, in which case our findings would be conservative. That said, the literature shows that although administrative data may not accurately capture the functional capacity of each individual patient, it does predict true diagnoses on average.^42^ Fourth, the HCUP severity of illness classification for mental health diagnoses does not measure the disease severity of individual patients, but instead classifies mental health diagnoses according to the strength of the diagnosis’ association with future hospitalization. Fifth, we only include in our study population those with a valid RLN to match multiple encounters across time and location, which may lead us to disproportionately exclude patients who are homeless or who cannot produce adequate identification. Sixth, we do not account for mortality in the time period studied because Vital Statistics data were unavailable when the analysis was conducted. We do not consider this limitation a threat to the validity due to low mortality rates among ED patients (0.3% of the overall population dies within 90 days of an ED visit, and 1.1% of frequent users).

## Conclusions

Among nonelderly adult patients presenting to California EDs in 2013, we identified several factors, observable based on ED records alone, that were associated with future ED utilization. We found that patients with more ED visits in the past year were also more likely to continue visiting the ED at high rates, but that other important clinical factors related to illness severity, particularly mental illness, were associated with substantial future ED use. With the exception of injury and poisoning diagnoses, the presence of a mental illness diagnosis did not appear to interact with other patient-level factors in a way that meaningfully alters associations with future ED use.

## Notes

Supplement. **eTable 1.** Comparison of Visits With and Without a Valid Record Linkage Number **eTable 2.** Healthcare Cost and Utilization Project Mental Health Severity Index, Reproduced From Coffey 2011 **eTable 3.** Logistic Regression Analysis (Sensitivity) **eTable 4.** Regression Sensitivity Analysis: 2+ HCUP Diagnoses Needed to Categorize Mild/Moderate/Severe
