# Supplementary material for: Factors Associated With Emergency Department Use by Patients With and Without Mental Health Diagnoses
Source: JAMA Netw Open. 2018 Oct 19;1(6):e183528. doi: 10.1001/jamanetworkopen.2018.3528 (PMC6324434; doi:10.1001/jamanetworkopen.2018.3528)
Supplement: Supplement. — eTable 1. Comparison of Visits With and Without a Valid Record Linkage Number eTable 2. Healthcare Cost and Utilization Project Mental Health Severity Index, Reproduced From Coffey 2011 eTable 3. Logistic Regression Analysis (Sensitivity) eTable 4. Regression Sensitivity Analysis: 2+ HCUP Diagnoses Needed to Categorize Mild/Moderate/Severe [file jamanetwopen-1-e183528-s001.pdf]

Supplementary Online Content

Niedzwiecki MJ, Sharma PJ, Kanzaria HK, McConville S, Hsia RY. Factors associated with emergency department use by patients with and without mental health diagnoses. *JAMA Netw Open*. 2018;1(6):e183528. doi:10.1001/jamanetworkopen.2018.3528

eTable 1. Comparison of Visits With and Without a Valid Record Linkage Number
eTable 2. Healthcare Cost and Utilization Project Mental Health Severity Index, Reproduced From Coffey 2011
eTable 3. Logistic Regression Analysis (Sensitivity)
eTable 4. Regression Sensitivity Analysis: 2+ HCUP Diagnoses Needed to Categorize Mild/Moderate/Severe

This supplementary material has been provided by the authors to give readers additional information about their work.

© 2018 Niedzwiecki MJ et al. *JAMA Network Open.*

eTable 1. Comparison of Visits With and Without a Valid Record Linkage Number

Variables	Proportion, Invalid RLN	Proportion, Valid RLN	Difference (Invalid - Valid)	P-value
Patient Characteristics				
Age 18 to 30	42.8%	30.2%	0.126	<0.01
Age 31 to 40	26.1%	20.1%	0.060	<0.01
Age 41 to 50	17.5%	20.9%	-0.034	<0.01
Age 51 to 64	13.6%	28.8%	-0.152	<0.01
Male	45.3%	43.9%	0.013	<0.01
White	21.6%	44.9%	-0.234	<0.01
Black	4.9%	14.4%	-0.096	<0.01
Hispanic	62.0%	30.6%	0.314	<0.01
Other Race	4.4%	4.5%	-0.002	<0.01
Unknown Race	7.2%	5.5%	0.017	<0.01
Privately Insured, Always	25.7%	38.3%	-0.126	<0.01
Medicare Insured, Ever	1.4%	10.1%	-0.087	<0.01
Medicaid Insured, Ever	31.3%	25.9%	0.054	<0.01
Uninsured, Ever	41.6%	25.7%	0.159	<0.01
Urban County of Patient Residence	93.9%	94.3%	-0.003	<0.01
Visit Characteristics				
Ever Admitted Inpatient	10.2%	12.2%	-0.020	<0.01
Any Primary MH Diagnosis	6.8%	6.7%	0.002	<0.01
Non-Mental Health CCS Codes (individual level)				
Infectious and Parasitic Diseases	7.1%	8.6%	-0.016	<0.01
Neoplasms	2.1%	3.8%	-0.017	<0.01
Endocrine; nutritional; and metabolic diseases and immunity disorders	14.6%	23.4%	-0.089	<0.01
Diseases of the blood and blood-forming organs	4.6%	6.3%	-0.017	<0.01
Diseases of the nervous system and sense organs	16.7%	21.7%	-0.050	<0.01
Diseases of the circulatory system	17.0%	27.3%	-0.103	<0.01
Diseases of the respiratory system	13.9%	18.9%	-0.050	<0.01
Diseases of the digestive system	14.4%	18.4%	-0.040	<0.01
Diseases of the genitourinary system	14.3%	15.7%	-0.014	<0.01
Complications of pregnancy; childbirth; and the puerperium	7.4%	4.5%	0.029	<0.01
Diseases of the skin and subcutaneous tissue	4.6%	6.5%	-0.019	<0.01
Diseases of the musculoskeletal system and connective tissue	10.6%	17.1%	-0.065	<0.01
Congenital anomalies	0.4%	0.6%	-0.002	<0.01
Certain conditions originating in the perinatal period	0.0%	0.0%	0.000	0.10
Injuries and poisoning	23.6%	22.0%	0.016	<0.01
Symptoms; signs; and ill-defined conditions and factors influencing health status	25.6%	29.3%	-0.037	<0.01
Residual codes; unclassified; all E codes	7.4%	11.6%	-0.042	<0.01
**N**	**875,110**	**6,698,794**		

© 2018 Niedzwiecki MJ et al. *JAMA Network Open.*

Notes: Visit level data from 2013. Patient's home zip code and county determined by the modal zip and county, respectively, over all visits.

eTable 2. Healthcare Cost and Utilization Project Mental Health Severity Index, Reproduced from Coffey 2011

Categories of M/SU disorders	ICD-9-CM Diagnosis Codes by Category and Severity Level
	**Severe**
Psychoses (not in NCS-R)	295(all); 297(all); 298(all)
Bipolar I and II conditions	296.00-06, 10-16, 40-46, 50-56, 60-66; 296.7; 296.80-82, 89, 90, 99
Drug dependence	304 (all); 648.3(all); 655.5(all); 760.72, 73, 75; 779.5; 965.0(all)
Obsessive-compulsive disorder	300.3
Dysthymia (chronic depression)	300.4; 309.1; 301.11-12
Oppositional defiant disorder	313.81
Related ICD-9-CM codes "severe"	296.20, 23, 24, 30, 33, 34; 301.20; 312.03, 13, 21; V11.0
	**Moderate**
Panic disorder	300.01, 21
Separation anxiety disorder	309.21
Attention deficit/hyperactivity conditions	314(all)
Agoraphobia without panic	300.22
Conduct conditions	312.00, 02, 10, 12, 20, 22; 312.4, 8, 9
Posttraumatic stress disorder	309.81
Drug abuse	292(all); 305.20-23, 30-33, 40-43, 50-53, 60-63, 70-73, 80-83, 90-93
Alcohol dependence	303.9(all); 357.5; 425.5; 535.3; 571.0, 1, 2, 3; V11.3
Any impulse control conditions	312.30-33, 39
Generalized anxiety disorder	300.00, 02, 09; 300.1(all); 300.20
Major depressive disorder (except for chronic depression, which is above)	296.22, 25, 32, 35; 311
	**Mild**
Social phobia	300.23
Alcohol abuse	291(all); 303.00-03; 305.00-03; V79.1; 790.3
Intermittent explosive disorder	312.34, 35
Any anxiety disorder (other anxiety)	300.23, 89; 300.5, 9; 308(all); 313.0, 1, 3; 313.21, 22, 82, 83
Any other disorder (other mental and substance-use disorders)	309.0; 309.22-24, 28, 29, 82, 83, 89; 309.3, 4, 9; 299(all); 307.3, 6, 7; 313.23, 89; 313.9; 307.20-23; 333.92;<br>301.0, 3, 4, 6, 7, 9; 301.10, 13, 21, 22, 50, 51, 59, 81-84, 89; 300.6, 7; 300.81, 82; 302.0, 1, 2, 3, 4, 6, 9;<br>302.50-53, 70-76, 79, 81-85, 89; 306.0, 1, 2, 3, 4, 6, 7, 8, 9; 306.50-53, 59; 307.40-49, 80, 81, 89; 648.4(all); V11.1, 2, 8, 9;<br>V15.4, V15.41, 42, 49; V40.2, 3, 9; V66.3; V67.3; V71.01, 02, 09; V79.0, 8, 9; 307.1; 307.50-54, 59
Specific phobia	300.29
Related ICD-9-CM codes specified "mild"	312.01, 11, 23; 296.21, 26, 31, 36; V65.42

Source: https://www.hcup-us.ahrq.gov/reports/SOI.jsp#data

© 2018 Niedzwiecki MJ et al. *JAMA Network Open.*

eTable 3. Logistic Regression Analysis (sensitivity)

Variables	OR (95% CI)
Patient Characteristics	
Age 31 to 40	0.93 (0.91,0.94)**
Age 41 to 50	0.85 (0.83,0.86)**
Age 51 to 64	0.70 (0.68,0.70)**
Black	1.37 (1.35,1.40)**
Hispanic	0.93 (0.92,0.94)**
Other Race	0.50 (0.49,0.52)**
Race Unknown	0.91 (0.89,0.94)**
Male	0.81 (0.80,0.82)**
Medicare Insured, Ever	2.35 (2.30,2.41)**
Medicaid Insured, Ever	2.68 (2.64,2.72)**
Uninsured, Ever	2.05 (2.02,2.07)**
Medium Poverty in Patient's Zip Code	1.21 (1.19,1.23)**
High Poverty in Patient's Zip Code	1.30 (1.27,1.32)**
Urban County of Patient Residence	0.89 (0.86,0.93)**
Psychiatrists per 10,000 Persons in Patient's County	0.95 (0.94,0.95)**
Visit Characteristics	
Frequent User (4-10 visits), Last Year	3.00 (2.93,3.08)**
Frequent User (11-17 visits), Last Year	5.19 (4.56,5.91)**
Frequent User (18+ visits), Last Year	22.76 (14.45,35.85)**
Admitted Inpatient	15.97 (15.63,16.33)**
Diagnoses	
Mental illness	1.75 (1.66,1.85)**
Mild MH Diagnosis (HCUP)	1.05 (1.03,1.06)**
Moderate MH Diagnosis (HCUP)	1.17 (1.16,1.19)**
Severe MH Diagnosis (HCUP)	1.30 (1.28,1.32)**
Primary MH Diagnosis	1.46 (1.44,1.49)**
Infectious and parasitic diseases	0.95 (0.93,0.97)**
Neoplasms	1.01 (0.98,1.04)
Endocrine; nutritional; and metabolic diseases and immunity disorders	0.92 (0.91,0.94)**
Diseases of the blood and blood-forming organs	0.67 (0.65,0.69)**
Diseases of the nervous system and sense organs	1.36 (1.34,1.38)**
Diseases of the circulatory system	1.06 (1.04,1.08)**
Diseases of the respiratory system	1.33 (1.32,1.35)**
Diseases of the digestive system	1.15 (1.14,1.17)**
Diseases of the genitourinary system	1.25 (1.23,1.27)**
Complications of pregnancy; childbirth; and the puerperium	0.93 (0.91,0.95)**
Diseases of the skin and subcutaneous tissue	1.39 (1.36,1.42)**
Diseases of the musculoskeletal system and connective tissue	1.40 (1.38,1.43)**
Congenital anomalies	0.87 (0.81,0.94)**
Certain conditions originating in the perinatal period	0.54 (0.30,0.96)*
Injury and poisoning	0.98 (0.97,1.0)*
Symptoms; signs; and ill-defined conditions	1.29 (1.27,1.31)**

© 2018 Niedzwiecki MJ et al. *JAMA Network Open.*

**eTable 3. Logistic Regression Analysis (sensitivity), continued**	
Interactions	
MH x Age 31 to 40	1.10 (1.07,1.13)**
MH x Age 41 to 50	1.09 (1.07,1.12)**
MH x Age 51 to 64	0.97 (0.94,0.99)*
MH x Male	1.05 (1.03,1.07)**
MH x Black	0.93 (0.91,0.95)**
MH x Hispanic	1.07 (1.05,1.09)**
MH x Other Race	1.31 (1.24,1.37)**
MH x Race Unknown	1.11 (1.07,1.15)**
MH x Medicare Insured, Ever	0.73 (0.71,0.76)**
MH x Medicaid Insured, Ever	0.76 (0.74,0.78)**
MH x Uninsured, Ever	0.81 (0.80,0.83)**
MH x Urban County of Patient Residence	0.96 (0.92,1.01)
MH x Medium Poverty in Patient's Zip Code	0.93 (0.91,0.95)**
MH x High Poverty in Patient's Zip Code	0.90 (0.88,0.93)**
MH x Admitted Inpatient	0.55 (0.53,0.56)**
MH x Psychiatrists per 10,000 Persons in Patient's County	1.04 (1.03,1.05)**
MH x Frequent User (4-10 visits), Last Year	0.63 (0.61,0.65)**
MH x Frequent User (11-17 visits), Last Year	0.56 (0.49,0.64)**
MH x Infectious and parasitic diseases	1.06 (1.03,1.09)**
MH x Neoplasms	0.89 (0.86,0.93)**
MH x Endocrine; nutritional; and metabolic diseases and immunity disorders	0.91 (0.89,0.94)**
MH x Diseases of the blood and blood-forming organs	1.10 (1.06,1.14)**
MH x Diseases of the nervous system and sense organs	1.00 (0.99,1.03)
MH x Diseases of the circulatory system	0.99 (0.96,1.01)
MH x Diseases of the respiratory system	0.93 (0.91,0.95)**
MH x Diseases of the digestive system	1.05 (1.03,1.07)**
MH x Diseases of the genitourinary system	0.92 (0.90,0.94)**
MH x Complications of pregnancy; childbirth; and the puerperium	1.21 (1.16,1.26)**
MH x Diseases of the skin and subcutaneous tissue	0.86 (0.84,0.89)**
MH x Diseases of the musculoskeletal system and connective tissue	0.98 (0.96,1.0)*
MH x Congenital anomalies	1.09 (0.99,1.19)+
MH x Certain conditions originating in the perinatal period	1.64 (0.82,3.29)
MH x Injury and poisoning	1.20 (1.17,1.22)**
MH x Symptoms; signs; and ill-defined conditions	1.13 (1.11,1.15)**
**N**	**3,446,338**

Notes: Logistic regressions with outcome variable equal to one if the patient is labeled a “Frequent user” with 4 or more visits in the one-year period following the index visit. Odds ratios reported. Diagnoses are grouped according to the Health Care Cost and Utilization Project’s Clinical Classification Software (CCS) and represent any diagnosis recorded in any visit to the emergency department (ED) or inpatient hospitalization following and ED visit. Statistical significance denoted by + (p<0.10), * (p<0.05), ** (p<0.01). Abbreviations: MH = mental health.

© 2018 Niedzwiecki MJ et al. *JAMA Network Open.*

eTable 4. Regression Sensitivity Analysis: 2+ HCUP Diagnoses Needed to Categorize Mild/Moderate/Severe

Variables	OR (95% CI)
Patient Characteristics	
Age 31 to 40	0.95 (0.94,0.95)**
Age 41 to 50	0.89 (0.89,0.90)**
Age 51 to 64	0.82 (0.81,0.83)**
Black	1.26 (1.25,1.27)**
Hispanic	1.00 (0.99,1.00)
Other Race	0.70 (0.69,0.70)**
Race Unknown	0.95 (0.94,0.96)**
Male	0.88 (0.88,0.89)**
Medicare Insured, Ever	1.81 (1.79,1.83)**
Medicaid Insured, Ever	1.94 (1.93,1.95)**
Uninsured, Ever	1.61 (1.60,1.62)**
Medium Poverty in Patient's Zip Code	1.11 (1.10,1.12)**
High Poverty in Patient's Zip Code	1.17 (1.16,1.17)**
Urban County of Patient Residence	0.92 (0.91,0.93)**
Psychiatrists per 10,000 Persons in Patient's County	0.96 (0.96,0.97)**
Visit Characteristics	
Frequent User (4-10 visits), Last Year	1.64 (1.62,1.66)**
Frequent User (11-17 visits), Last Year	1.97 (1.86,2.08)**
Frequent User (18+ visits), Last Year	5.91 (5.00,6.98)**
Primary MH Diagnosis	1.31 (1.30,1.33)**
Admitted Inpatient	4.88 (4.83,4.93)**
Diagnoses	
Mental illness	1.38 (1.34,1.41)**
2+ Mild MH Diagnoses (HCUP)	1.24 (1.22,1.26)**
2+ Moderate MH Diagnoses (HCUP)	1.36 (1.34,1.37)**
2+ Severe MH Diagnoses (HCUP)	1.24 (1.22,1.26)**
Infectious and parasitic diseases	1.00 (1.00,1.01)
Neoplasms	1.04 (1.03,1.05)**
Endocrine; nutritional; and metabolic diseases and immunity disorders	0.99 (0.99,1.00)*
Diseases of the blood and blood-forming organs	0.83 (0.82,0.84)**
Diseases of the nervous system and sense organs	1.22 (1.21,1.22)**
Diseases of the circulatory system	1.06 (1.05,1.06)**
Diseases of the respiratory system	1.22 (1.21,1.23)**
Diseases of the digestive system	1.13 (1.12,1.14)**
Diseases of the genitourinary system	1.19 (1.18,1.19)**
Complications of pregnancy; childbirth; and the puerperium	1.04 (1.03,1.05)**
Diseases of the skin and subcutaneous tissue	1.33 (1.32,1.34)**
Diseases of the musculoskeletal system and connective tissue	1.25 (1.24,1.25)**
Congenital anomalies	0.98 (0.95,1.00)+
Certain conditions originating in the perinatal period	0.98 (0.82,1.18)

© 2018 Niedzwiecki MJ et al. *JAMA Network Open.*

Injury and poisoning	0.98 (0.98,0.99)**
Symptoms; signs; and ill-defined conditions	1.17 (1.16,1.17)**
**eTable 4. Regression Sensitivity Analysis: 2+ HCUP Diagnoses Needed to Categorize Mild/Moderate/Severe, continued**	
Interactions	
MH x Age 31 to 40	1.06 (1.04,1.07)**
MH x Age 41 to 50	1.04 (1.03,1.06)**
MH x Age 51 to 64	0.95 (0.94,0.97)**
MH x Male	1.04 (1.03,1.05)**
MH x Black	0.94 (0.93,0.95)**
MH x Hispanic	1.01 (1.00,1.02)
MH x Other Race	1.10 (1.08,1.12)**
MH x Race Unknown	1.06 (1.04,1.08)**
MH x Medicare Insured, Ever	0.85 (0.84,0.86)**
MH x Medicaid Insured, Ever	0.87 (0.86,0.88)**
MH x Uninsured, Ever	0.92 (0.91,0.93)**
MH x Urban County of Patient Residence	1.01 (0.99,1.03)
MH x Medium Poverty in Patient's Zip Code	0.97 (0.96,0.98)**
MH x High Poverty in Patient's Zip Code	0.96 (0.95,0.97)**
MH x Admitted Inpatient	0.68 (0.67,0.69)**
MH x Psychiatrists per 10,000 Persons in Patient's County	1.02 (1.01,1.03)**
MH x Frequent User (4-10 visits), Last Year	0.66 (0.65,0.66)**
MH x Frequent User (11-17 visits), Last Year	0.59 (0.55,0.62)**
MH x Frequent User (18+ visits), Last Year	0.47 (0.40,0.56)**
MH x Infectious and parasitic diseases	1.01 (1.00,1.02)
MH x Neoplasms	0.93 (0.91,0.94)**
MH x Endocrine; nutritional; and metabolic diseases and immunity disorders	0.92 (0.91,0.93)**
MH x Diseases of the blood and blood-forming organs	0.99 (0.98,1.01)
MH x Diseases of the nervous system and sense organs	1.03 (1.03,1.04)**
MH x Diseases of the circulatory system	0.98 (0.97,0.99)**
MH x Diseases of the respiratory system	0.95 (0.94,0.96)**
MH x Diseases of the digestive system	1.01 (1.00,1.02)*
MH x Diseases of the genitourinary system	0.94 (0.93,0.95)**
MH x Complications of pregnancy; childbirth; and the puerperium	1.06 (1.04,1.08)**
MH x Diseases of the skin and subcutaneous tissue	0.87 (0.86,0.89)**
MH x Diseases of the musculoskeletal system and connective tissue	1.00 (0.99,1.01)
MH x Congenital anomalies	1.03 (0.98,1.07)
MH x Certain conditions originating in the perinatal period	0.91 (0.71,1.17)
MH x Injury and poisoning	1.13 (1.12,1.14)**
MH x Symptoms; signs; and ill-defined conditions	1.10 (1.09,1.11)**
**N**	**3,446,338**

© 2018 Niedzwiecki MJ et al. *JAMA Network Open.*

Notes: Negative binomial regression analysis (log link function) with an outcome variable indicating the total count of ED visits in the 365 days following the index visit. Results are reported as incidence rate ratios (IRRs). Two or more HCUP diagnoses required to classify severity, e.g. 2 or more instances of a “severe” mental health diagnosis required to classify as “severe.” Diagnoses are grouped according to the Health Care Cost and Utilization Project’s Clinical Classification Software (CCS) and represent any diagnosis recorded in any visit to the emergency department (ED) or inpatient hospitalization following and ED visit. The omitted diagnosis group is residual codes, unclassified, and E Codes (CCS category 18). Abbreviations: MH = mental health. Statistical significance denoted by + (p<0.10), * (p<0.05), ** (p<0.01).

© 2018 Niedzwiecki MJ et al. *JAMA Network Open.*
